# Theory-driven, web-based, computer-tailored advice to reduce and interrupt sitting at work: development, feasibility and acceptability testing among employees

**DOI:** 10.1186/s12889-015-2288-y

**Published:** 2015-09-24

**Authors:** Katrien De Cocker, Ilse De Bourdeaudhuij, Greet Cardon, Corneel Vandelanotte

**Affiliations:** Department of Movement and Sports Sciences, Ghent University, Ghent, Belgium; Research Foundation Flanders, Brussels, Belgium; Physical Activity Research Group, School for Human Health and Social Sciences, Central Queensland University, Rockhampton, QLD Australia

**Keywords:** Sedentary behaviour, Computer-tailoring, Employees, e-health

## Abstract

**Background:**

Because of the adverse health effects in adults, interventions to influence workplace sitting, a large contributor to overall daily sedentary time, are needed. Computer-tailored interventions have demonstrated good outcomes in other health behaviours, though few have targeted sitting time at work. Therefore, the present aims were to (1) describe the development of a theory-driven, web-based, computer-tailored advice to influence sitting at work, (2) report on the feasibility of reaching employees, and (3) report on the acceptability of the advice.

**Methods:**

Employees from a public city service (n = 179) were invited by e-mail to participate. Employees interested to request the advice (n = 112) were sent the website link, a personal login and password. The online advice was based on different aspects of the Theory of Planned Behaviour, Self-Determination Theory and Self-Regulation Theory. Logistic regressions were conducted to compare characteristics (gender, age, education, employment status, amount of sitting and psychosocial correlates of workplace sitting) of employees requesting the advice (n = 90, 80.4 %) with those who did not. Two weeks after visiting the website, 47 employees (52.2 %) completed an online acceptability questionnaire.

**Results:**

Those with a high education were more likely to request the advice than those with a low education (OR = 2.4, CI = 1.0-5.8), and those with a part-time job were more likely to request the advice compared to full-time employees (OR = 2.9, CI = 1.2-7.1). The majority found the advice interesting (n = 36/47, 76.6 %), relevant (n = 33/47, 70.2 %) and motivating (n = 29/47, 61.7 %). Fewer employees believed the advice was practicable (n = 15/47, 31.9 %). After completing the advice, 58.0 % (n = 25/43) reported to have started interrupting their sitting and 32.6 % (n = 17/43) additionally intended to do so; 14.0 % (n = 6/43) reported to have reduced their sitting and another 51.2 % (n = 22/43) intended to do so.

**Discussion:**

More efforts are needed to reach lower educated and full-time workers. Further research should examinethe effects of this intervention in a rigorous randomised controlled trial.

**Conclusions:**

It is feasible to reach employees with this tool. Most of the employees who requested the advice found itacceptable and reported they changed their behaviour or intended to change it. Interrupting sittingappeared more achievable than reducing workplace sitting.

## Background

Levels of sedentary behaviour (i.e. activities in a sitting or reclining posture characterized by a low energy expenditure [1]) are high in modern societies [2–4]. Especially in the workplace, individuals spend a lot of time sedentary [5]. For example, full-time employed Australian adults spent on average 6.8 h/day sitting at work [6] and US employees in sedentary occupations sat for 11 h/day [7]. Recent evidence showed that both the total amount of sedentary time and the pattern of sedentary behaviour (i.e. prolonged uninterrupted periods) were associated with several adverse health effects in adults, e.g. obesity, metabolic syndrome, type 2 diabetes, some cancers, and all-cause and cardio-vascular disease mortality [5, 8–10], independent of other factors such as body weight, diet and physical activity [1]. For example, the negative health impact of sedentary behaviour even occurs in those meeting the health-related guideline for physical activity (150 min of moderate-intensity aerobic physical activity a week) [11]. Therefore, interventions to reduce (limit the amount of sitting) and/or interrupt (limit prolonged sitting bouts) sitting at work are needed [12].

Today, a limited number of interventions have been developed aimed at reducing or interrupting sitting at work. The used strategies in these workplace interventions were a variety of approaches including changes in the environmental level such as the use of (passive) prompting software [13–15] and (shared) sit-to-stand workstations [16–18], or a combination of organisational-, individual- and/or environmental level components [19–23]. Examples of strategies at the organisational level are senior management consultation, representatives consultation workshop, team champions, staff information and brainstorming sessions, and supportive emails from managers to staff. At the individual level, face-to-face coaching sessions, telephone support, the use of an educational package (with a multimedia presentation, self-monitoring of breaks from sitting and visual and auditory reminders about taking breaks from sitting) can be implemented. Findings of completed effect studies [13–19, 22] suggest that these interventions can be effective in changing sitting at work, however most of these interventions had a very limited reach. So the evidence on the effectiveness of each and every single strategy is still scarce and our understanding of how to best influence workplace sitting is yet limited [24, 25]. Moreover, some (effective) intervention strategies, including sit-to-stand workstations and face-to-face sessions are costly to implement, suggesting that inexpensive alternative strategies are warranted. Consequently, in the future, intervention strategies that can effectively and affordably reach large numbers of employees are needed. In order to avoid the implementation of effective and affordable strategies that do not apply to the target population and to determine whether an intervention is appropriate for further effectiveness testing, studies that examine the feasibility and acceptability of such approaches [26, 27] are first needed.

One intervention approach, currently being used by public health promoters, that is found to be feasible, acceptable, and successful in changing a variety of health-related behaviours (including dietary behaviours, alcohol consumption, smoking habits and physical activity), is computer-tailoring via the Internet [28–31]. Interactive web-based interventions create the opportunity for on-going contact with and support to its respondents, and use tools that support self-regulatory skills, such as goal setting activities, self-monitoring tools, skill building activities, email reminders, booster sessions, and interactive activities [32, 33]. Despite the advantages (low cost, no limitations due to time or location, two billion Internet users worldwide [34]) no web-based computer-tailored interventions targeting workplace sitting were found in the literature. As the promising effects of tailoring for other health-related behaviours may be behaviour specific [32, 33], research regarding computer-tailored interventions for sitting time in a variety of settings is needed.

Therefore, a web-based, computer-tailored intervention aimed at reducing sitting at work was developed. Based on the abovementioned literature, we aimed to develop an interactive intervention [32, 33] that integrates self-management (e.g. goal-setting and action planning) [32, 33]. In addition, we aimed to include tailoring-constructs based on a health behaviour theory, as a review showed that theory-based computer-tailored interventions reported more positive outcomes compared to non-theory-driven interventions [35]. Important aspects in the process of intervention development are the evaluation of the feasibility (What is the ability to reach employees with the intervention? Who is requesting the advice?) and acceptability (How do those requesting the advice evaluate the intervention?) of potential intervention strategies in particular contexts and target groups [26, 27]. This can prevent implementing an intervention that does not appeal to the target population.

The first objective of the present paper was to describe (the development of) this theory-driven, web-based, computer-tailored advice. The second objective was to report on the feasibility of reaching employees by comparing characteristics of employees taking part in the intervention with those who did not. Finally, the third objective was to report on the acceptability of the advice among those who participated. The overall research question was ‘Is a theory-driven, web-based, computer-tailored intervention feasible and acceptable for employees to reduce or interrupt workplace sitting?’.

## Methods

### Development of the theory-driven, web-based, computer-tailored intervention

The general approach and theoretical background of this intervention was based on our previously developed computer-tailored physical activity interventions [36–38]. In the present web-based intervention, called ‘*start to stand’*, users received personalized computer-tailored advice on their sitting, including tips and suggestions on how to interrupt (having standing breaks) and reduce (replacing sitting by periods of standing) sitting. To receive the computer-tailored advice, users had to log onto a website using a confidential username and password (this was being e-mailed by the researchers). After logging-in, users received a short introduction pop-up screen and were then referred to the home page, inviting them to complete an assessment questionnaire. A set of decision rules (i.e. pre-established computer algorithms defined by the researchers) selected the feedback messages that were matched and tailored to the specific answers of the user [39]. The combination of these feedback messages formed the tailored advice that appeared immediately on the user’s screen after completing the assessment questionnaire and could be printed.

At the end of the advice (here called ‘section 1’, see further), users were invited to request up to 5 other non-committal sections if they were interested. They were referred to the homepage on which the links to the different sections appeared simultaneously. The focus was respectively on sitting during work hours (section 2: standing interruptions, section 3: replacing sitting by standing), during commuting to work (section 4) and during (lunch) breaks at work (section 5); or on making an action plan (section 6).

Throughout the advice, the tailoring-constructs were based on the *Theory of Planned Behaviour* (*TPB*) [40]. The *TPB* focusses on the intention (or motivation) to adopt or modify a behaviour. This theory assumes that the intention is higher when people have more positive attitudes, perceived social influence and self-efficacy (see further for content of the advice). In our previously developed and effective computer-tailored interventions, the feedback was also based on the *TPB*. However, the literature suggests to expand the constructs of the *TPB* and increased attention has been given to ‘post-motivational’ constructs, such as concepts of ‘self-regulation’ [41]. As a result, it was decided to integrate the concept of goal setting in the advice (see section 6 for further details). In addition, it has been argued that not only the amount of intention or motivation matters, but also the type of it [41]. Based on the suggestion to integrate assumptions from the *Self-Determination Theory* (*SDT*) [42, 43] with those of socio-cognitive models (*TPB*) [41], concepts of the *SDT* were taken into account during the development of the present advice. In line with the *SDT* [42, 43], users were able to decide which section of the advice they requested (after receiving the general section of the advice), instead of forcing participants to answer all assessment questions at once and providing a much longer advice containing all information. Leaving a choice to the users to decide what and when should be requested is assumed to increase autonomous motivation of the user, which is more likely to lead to behavioural change [42, 43]. The *SDT* already showed good evidence of its value in research on health-related behaviours such as exercise and physical activity [44] and some recent web-based computer-tailored physical activity interventions are being based on the *SDT* [45].

Throughout all sections a progression indicator at the top of the internet page showed how many assessment questions (%) needed to be completed before receiving the advice. The advice of all sections contained pictures related to the content being discussed. A reminder system was developed, automatically e-mailing logged-in users when they did not fully complete the assessment questionnaire of section 1 or when they initially did not request any other additional sections. Up to 3 reminders were sent with a 7 day-interval. Users could regulate this system in a separate tab page on the website and change the number of reminders and the interval period. The opportunity to manage the reminder e-mail system is an approach also based on the *SDT*, increasing autonomy of the user. Below and in Table 1, an overview of the assessment questionnaires, the structure and content of the advice and the theoretical basis is provided for every section.

**Table 1:** Structure and content of the online questionnaire and computer-tailored advice of the different sections

Assessment questionnaire (*answering options*)	Feedback (number of different tailored feedback messages)
	*Example of feedback message*
Section 1: General advice on sedentary behaviour (SB)	
• Demographics: *-* first name, last name (*open ended*) *-* sex (*male/female*) *-* age (*<18 years, 18, … 65, >65 years*) • Height (*<145, 145,… 210, 210 cm*) and weight (*<40, 40, … 120, > 120 kg*) • Number of workdays in a usual week (*1*–*7*) • Job tasks (yes/no): *-* phone calls, *-* computer work, *-* desk work, *-* meetings, *-* visiting clients • Knowledge about SB (*disagree/unsure/agree*) *-* link total SB with health *-* independence of physical (in)activity *-* link interruptions in SB with health • Workforce Sitting Questionnaire (*hours and minutes*): SB during transport, work, TV, PC use at home, leisure time on work days and non-work days • Interruptions every 30 minutes during prolonged SB (*rarely/sometimes/mostly*) • International Physical Activity Questionnaire short version (*days, hours and minutes*): *-* walking *-* moderate intensity physical activity *-* vigorous intensity physical activity	• Information: why focus on SB? (generic) *Advice on sitting and standing may seem a bit weird at first. Sitting is a habitual behaviour, we are not aware of doing it, but still it is import as too much sitting is bad for your health.* • Feedback (including graph) about *-* total SB: 4 categories (<8, <12, <16 and ≥16 hrs/day) (18 different messages) *-* SB on work day and non-work day (36 different items) *On average you sit 15.1 hours per day, which is more than the recommended maximum of 8 hours per day. As you already suspected, your level of SB can cause health problems.* • Information about why interruptions are important (generic) + feedback about user’s interruptions (9 different messages) *Research shows that an interruption in prolonged sitting, for example every 30 minutes, can be sufficient to counter the unhealthy processes of sitting for long periods. You indicated that you interrupt your sitting sometimes. This is positive, however try to increase this in the future…* • Information about the relationship between physical activity and SB (generic) + feedback about user’s physical activity (12 different messages) *Prolonged sitting is associated with increased health risks, even if you are regularly being physically active. So, regardless of how much you move, too much sitting is bad. You indicated that you are on average moderately active for 10 minutes a day. This is not enough to meet the health-related physical activity guidelines, indicating that you need 30 minutes of moderate-to-vigorous physical activity every day to improve your health*. • Links to the other sections of the website (generic): *What now? Okay, you are at the end of the general section of this ‘start to stand’-advice. In the other sections you will get specific tips and suggestions regarding your SB at work. You have the choice to request the other sections now or later. In each section you will have to complete some questions and based on your answers, a personal advice will be provided. Find it all out on the homepage…*
Section 2: Standing interruptions in SB during work hours (non-committal)	
• Frequency (*number per x hour*) and duration (*<1’/≥1’*) of standing interruptions • Attitudes about standing up every 30 minutes (*disagree/unsure/agree*): *-* stupid, healthy, annoying, relaxing, tiring • Self-efficacy for standing interruptions (*disagree/unsure/agree*): *-* overall *-* when having no social support • Barriers for standing interruptions (*select*): stress, meetings, tired legs, busy day, uncomfortable shoes,… • Intention to stand up every 30 minutes (*no/within 6 months/within 1 month*) • Autonomy to decide to stand up every 30 minutes (*disagree/unsure/agree*)	• Feedback about interrupting behaviour (15 different messages) *You indicated that you interrupt your sitting at work once every two hours for at least one minute. Research shows that one interruption every 30 minutes for one minute is sufficient to counter the unhealthy effects of prolonged sitting. So it is positive that you stand for at least one minute, but you need to do this more frequently as you are sitting too long. The tips below can help you…* • Feedback about attitudes, intentions and social support for interruptions (30 different items) *You mentioned you are intending to stand up regularly at work within one month from now. That’s excellent! You also think positive about these interruptions. That’s why this advice is definitely for you and it will help you with this challenge.* • Tips to increase/maintain interruptions tailored to job tasks (15 different messages) *You mentioned you don’t have to get up to take your prints. Why not put your printer further away from your desk so that you need to get up and interrupt your sitting. You could also decide to put other devices such as the coffee machine out of reach!* • Tips on how to overcome barriers (8 different messages) *You think you won’t be able to interrupt your sitting when you are being busy. The good news is that changing your SB doesn’t need to take long. Small changes like getting up when the phone rings or reading some documents while standing,… are easier to implement than you might think.* • Feedback about self-efficacy and social support (9 different messages) *A lack of support from the management doesn’t seem to bother you to stand up regularly. It is very positive that you’re confident that you can stand up even if they think this is weird. Keep up this spirit! Though, it’s a pity you doubt you can stand up when colleagues don’t support you or think this is ridiculous. Maybe they are not familiar with the concept of ‘stand more, sit less’. Try to convince them that standing up regularly is meaningful!* • Feedback about the autonomy to make decisions (3 different messages) *Finally, you have, to a certain degree, autonomy over the decision to stand up during work hours. Take advantage of this privilege. Good luck with the implementation of the tips!* • Invitation to other sections (generic) *If you want to design a concrete personal plan to change your SB at work, click [here].*
Section 3: Replacing SB by standing during work hours (non-committal)	
• Job roster (*half-time, half days/half-time, full days/full-time*) • Job hours (only for full-time workers) (*<42 hours/week/≥42 hours/week*) • Attitudes about (partly) standing during work (*disagree/unsure/agree*): *-* stupid, healthy, annoying, relaxing, tiring • Self-efficacy for standing during work (*disagree/unsure/agree*): *-* overall *-* when feeling tired, when feeling stressed • Advantages of standing during work (*select*): more focus, stronger legs, better energy balance,… • Intention to stand during work (*no/within 6 months/within 1 month*) • Autonomy to decide to stand during work (*disagree/unsure/agree*)	• Feedback about SB during working hours tailored to job roster and job hours: 3 categories based on <180, <342, <378 minutes/day (24 different messages) *It appears that you spend 420 minutes (about 7 hours) sitting during work hours. This is too much, as this is more than 75 % of the time you work. So you sit more than other employees do. You mentioned you work overtime a lot, this is probably the cause for your prolonged sitting. In this case, reducing these long periods of sitting is even more important! Try to sit less at work every day. This is easier than it seems and you can for example do this in small blocks, once in the morning and once in the afternoon.* • Feedback about attitudes and intention to stand during work (40 different messages) *You mentioned you are not intending to stand while working. It’s a pity, but maybe the following tips can change your mind. We invite you to read them, even though you are not positive about standing during work hours. Give it a chance and maybe you experience some benefits while trying. Good luck!* • Tips to increase/maintain standing tailored to job task (7 different messages) *Check whether you have the ability to put your laptop or computer on a raised table or cupboard. It is not necessary to stand the whole day, but choosing to stand for a couple of hours is an excellent idea! Build this up slowly and start with short periods of standing. You’ll get used to it quickly.* • Feedback on benefits (6 different messages) *An important benefit of standing during work hours for you is a better health in the long-term. Too much sitting is indeed associated with a higher risk for diabetes, cancer, heart disease and mental illness. Furthermore, there is convincing evidence that sedentary behavior (= sitting) is linked to increased mortality in men and women. So it is a great idea to sit less and stand more.* • Feedback about what is stimulating, tailored to self-efficacy (9 different messages) *You don’t mind to stand even when you are tired. This is wonderful, because reducing your sitting and standing while working will give you more energy compared to staying seated the whole day.* • Feedback about the power to make decision (3 different messages) *Finally, you have the autonomy to decide to stand while working. Take advantage of this privilege. You will see that it can often be easy to change your sedentary behaviour. Good luck with the implementation of the tips!* • Invitation to other sections (generic) *Take also a look at the other sections to get a complete personalized ‘start to stand’-advice!*
Section 4: SB during commuting to work (non-committal)	
• Public transport (*yes/no*) • Distance to transport stop or work (*<2/2–8/>8 km*) • Attitudes about standing during public transport or active transportation (*disagree/unsure/agree*): *-* stupid, healthy, annoying, relaxing, tiring • Self-efficacy for standing during public transport or active transportation (*disagree/unsure/agree*): *-* overall *-* when having no social support • Barriers for active transportation (*select*): weather, traffic, lack of time,… • Ability to change current transport mode given living situation (*disagree/unsure/agree*)	• Feedback about transport mode + tips to change (28 different messages) *You mention you generally use public transport to go to work. This is great because this means you are able to stand during the trip. We recommend you to stand during at least 10 minutes of your trip in order to reduce your amount of sitting. Please also stand while you are waiting at the stops.* • Feedback about attitudes and self-efficacy for standing during public transport/active transportation (47 different messages) *You travel by car, but you have the choice to park your car further away and walk the last 5 or 10 minutes. This is a great opportunity to increase your physical activity. You also feel positive about this, but you believe you are not able to achieve this challenge. Maybe this advice will change your way of thinking because the effort is smaller than you imagine.* • Tips to overcome barriers (32 different messages) *A lack of time is an important barrier for you to park your car further away and walk the last part. Realize this is only 5 to 10 minutes of your entire day… in addition, walking is good for your health, fitness and weight!* • Feedback about the ability to change transport mode (9 different messages) *Finally, you believe that in your situation it is too hard to park your car further away and walk some part. This is a pity. Maybe this advice can change your mind and make you more open-minded to active transportation. If not, the information in the other sections may be important for you.* • Invitation to other sections (generic) *You’re at the end of this section. Also have a look at the other sections…*
Section 5: SB during (lunch) breaks at work (non-committal)	
• Duration lunch break *(<30 min/30–60 min/> 1 hour*) • Type of meal during lunch break (*sandwiches/hot meal*) • Number of lunch breaks spent (*1*–*5*): *-* sitting *-* actively • Attitudes about changing SB during (lunch) breaks (*disagree/unsure/agree*): *-* stupid, healthy, annoying, relaxing, tiring • Self-efficacy for changing SB during (lunch) breaks (*disagree/unsure/agree*): *-* overall *-* when having no social support *-* when being busy	• Feedback about how breaks from work are being spent (18 different messages) *You mention you spend your lunch break and coffee breaks mostly sitting…* • Feedback about attitudes and self-efficacy for changes in SB during breaks (27 different messages) *…You believe you’re not able to change this. However, these breaks provide a unique opportunity to sit less (or be active), especially when this is difficult during the work hours. Try at least to stand for half of your breaks. By trying you may change your mind about what is possible…* • Tips to change (13 different messages) *As you eat sandwiches during lunch, you are able to eat these while standing or walking. Even though there are no raised tables available, check whether it is possible to stand in the canteen. A sandwich meal can even be taken while walking, in the fresh air.* • Invitation to other sections (generic)
Section 6: action plan: plan to change SB (non-committal)	
• What do you want to change? (*increase interruptions/replace sitting by standing/both*) • How often, how long? • In which situations do you want to make changes? (*work hours/work breaks/commuting/combinations*) • Which actions seem feasible? (*select + open-ended*) e.g. if the phone rings, then I will stand up; if my computer starts up, then I will install a software program to remind me to stand up every x minutes; if I’m waiting for the bus/train, then I will stand; if I restart work after lunch time, then I will work standing for a period;…	• What will I change? (3 different messages) *I will increase my interruptions in sitting by standing up frequently* • How long/how often will I do this? (12 different items) *Every 45 minutes, I will stand up for 40 seconds* • In which situations will I do this? (7 different messages) *During the working hours, work breaks and commuting to/from work* • IF-THEN plan (25 different messages) - *if I have a meeting, I will get something to drink halfway the meeting* *- if I need to put something in the bin, I will stand up* *- if I eat a snack at my desk, I will stand up* *- if I have a glass of water, I will stand up while drinking* *- if I have a hot meal during lunch, I will clean up immediately instead of staying seated at the table.* • When will I start doing this? (individual) *Monday March 10, 2014*

*SB* sedentary behaviour

#### Section 1: General advice on sedentary behaviour

##### Assessment questionnaire

The questionnaire included demographic factors, height and weight, job-related information, knowledge about sedentary behaviour, level of sitting time in five domains (see Workforce Sitting Questionnaire in the ‘measures’ section [46]), frequency of interruptions in prolonged sitting, and level of physical activity (International Physical Activity Questionnaire short version [47, 48] (see Table 1, part 1). Depending on the answers given, a maximum of 37 questions needed to be completed.

##### Advice

Users first received general information on why it is important to focus on sedentary behaviour, in order to increase their knowledge on this topic [49]. Knowledge at some level is a logical requirement for the intentional performance of health-related behaviours [50]. This was followed by normative feedback on their own amount of sitting on working and non-working days, which was related to four categories, being: <8 h per day, <12 h per day, <16 h per day, and ≥16 h per day- mainly based on research confirming the association of sedentary behaviour with all-cause mortality [51]. The aim was to increase awareness of users’ levels of sitting time. Further, feedback on the frequency of interruptions, information on the importance of interruptions [2] and the suggestion to interrupt prolonged sitting every 30 min [21, 52] were given. Also the negative health impact of too much sitting, independent of physical inactivity [8], was explained. Again, this was done in order to increase knowledge and awareness regarding sedentary behaviour. Next, feedback on users’ physical activity level was given. Examples of the feedback messages are provided in Table 1. Finally, users were encouraged to request the other non-committal sections.

#### Sections 2–5

The structure and content of the questionnaires and the advice itself of the additional sections (2: standing interruptions in sitting at work; 3: replacing sitting at work by standing; 4: sitting during commuting; 5: sitting during (lunch) breaks at work) were similar to the first section of the advice (see Table 1).

##### Assessment questionnaires

Maximum 19 questions per section were used to assess users’ current behaviours and psychosocial correlates (attitudes, self-efficacy, social support, perceived benefits and barriers, intentions) of sitting behaviours (see Table 1). The questions about these constructs of the *TPB* [40] were based on previously validated questions [53] and used in studies assessing psychosocial correlates of sedentary behaviour [54, 55]. For attitudes towards the different behaviours, 5 items were used (stupid, healthy, annoying, relaxing, tiring). Self-efficacy towards interrupting or reducing sitting was asked using at least two items in each section [overall self-efficacy (section 2–5) and self-efficacy when having no social support (section 2, 5)/when feeling tired or stressed (section 3)/when being busy (section 4, 5)]. For these items users could agree or disagree with the statements or indicate they were unsure about it. For the perceived benefits and barriers, users could select up to two items for both constructs (see Table 1 for details). The items on intentions could be answered with ‘no intention to change’ , ‘intending to change within 6 months’ , or ‘intending to change within 1 month’. Finally, users were asked whether they had power to decide (autonomy) to interrupt or reduce sitting (1 item, disagree/unsure/agree).

##### Advice

Within each part, the advice appeared immediately on the computer screen. Users were given feedback about their attitudes, self-efficacy, social support, knowledge, perceived benefits and barriers and intentions related to reducing/interrupting sitting. More details about each section and examples of the feedback messages are provided in Table 1.

#### Section 6: Action plan to change sitting behaviour

As goal setting is a common and effective intervention technique used in other health behavioural change programs [48], it was decided to include an ‘action plan’ in the present intervention as well. The action plan operates within the *Self-Regulation Theory* (*SRT*) [56]. The action plan was meant for users who were motivated to change their sitting and aimed to convert intentions into specific actions, through SMART (Specific, Measurable, Attainable, Relevant and Time-bound) goals and implementation-intentions. Users were able to formulate these goals themselves instead of providing compulsory recommendations. Consequently, intentions or goals were set by and not for the users [56]. Specific goals are assumed to be more effective than general ‘do-your-best’ goals. The aim is to transform intentions into specific actions and to start a thought process which directs people on how to become less sedentary (implementation-intentions) [57]. An action plan was also used in web-based computer-tailored interventions promoting physical activity [36, 37] and the formation of implementation-intentions has also demonstrated to enhance the occurrence of other health-related behaviours, such as breast self-examination, healthy eating, and cervical cancer screening [56].

##### Assessment questionnaire

Users were asked *what* (reduce or interrupt sitting or both) they want to do, *how long*, *how often*, *when* and *how* in order to state personally relevant and attainable goals. Finally, users were able to select pre-composed ‘if-then’-statements in order to make an ‘if-then’ plan. As the matter of sedentary behaviour is fairly new at this stage [58], it was decided to provide examples of ‘if-then’-statements (see Table 1). Users were also able to formulate ‘if-then’-statements themselves in an open-ended question format. A maximum of 12 questions needed to be completed.

##### Advice

When these questions were completed, a schematic overview of this information was immediately provided on the screen (see Table 1 for example).

### Study design and study sample of the feasibility and acceptability testing

This descriptive study tested the feasibility and acceptability of the intervention in a quantitative way. Participants were recruited from a public city service (located in Flanders, northern part of Belgium) that was enrolled as control condition in a previous intervention study targeting sitting at work, conducted in November 2012. Employees participating then (*n* = 188) were considered eligible for the present study. Participants completed an informed consent form and the study protocols were approved by the Ethics Committee of the Ghent University Hospital, Belgium.

### Procedures of the feasibility and acceptability testing

As part of the previous study (November 2012), participants completed a questionnaire on socio-demographics, levels of sitting time, and psychosocial correlates of sitting at work (see further). The questionnaire was sent by e-mail and participants could complete either a digital version or print the questionnaire and complete a paper version. The completed questionnaire was then sent back (e-mail) to the researchers.

**Fig. 1:**
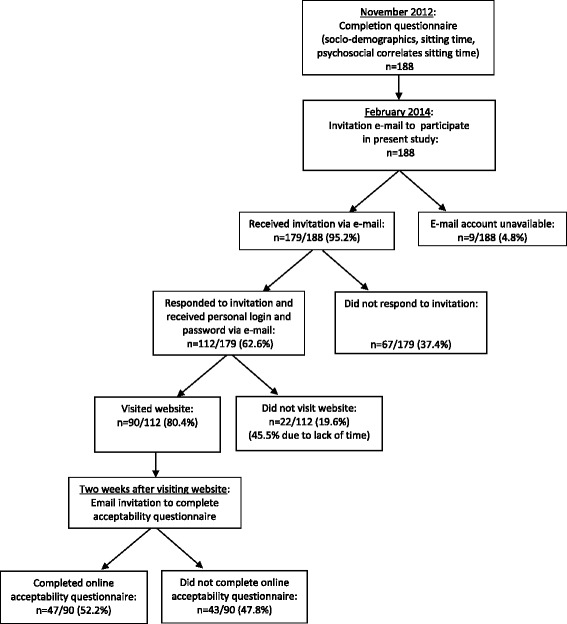
Flow chart of the study

In February 2014, employees were sent an e-mail inviting them to request web-based, computer-tailored advice about sitting. Interested employees were asked to reply to the e-mail and were then sent a link to the website, a personal log-in and password to enter the website. Researchers kept track of those who had logged onto the website. Two weeks after visiting the website, an e-mail with a link to an online questionnaire (QuestionPro, mean duration to complete: 7.9 ± 6.5 min) was sent to invite employees who requested the advice to participate in the acceptability testing. Participants who were initially interested but who did not request the advice within two weeks, were sent a reminder to visit the website. Two weeks after this reminder, an e-mail was sent to those who still did not visit the website asking them why they did not request the advice (‘no interest’ , ‘no time’ , ‘I intended to, but forgot about it’ , ‘the link to the website was not working’ , ‘I didn’t manage to log on’ , ‘the website was too slow’ , ‘there were too many questions’ , ‘it was too complicated for me’). A flow chart of the study is presented in Fig. 1.

### Measures used for the feasibility and acceptability testing

#### Questionnaire on socio-demographics, levels of sitting time and psychosocial correlates of sitting at work (background information used for the feasibility testing)

This questionnaire was completed as part of the previous study and was not reassessed in 2014. Participants were asked to provide their sex, age (open-ended), education (no diploma, lower secondary, secondary, college, university), and employment status (full-time or part-time employed). Education was dichotomized into low education (no diploma, lower secondary, and secondary) and high education (college and university).

The Workforce Sitting Questionnaire (WSQ) was used to measure sitting time [46]. This questionnaire assesses time spent sitting on a workday and a non-workday for the last seven days while (1) travelling to and from places; (2) being at work; (3) watching TV; (4) using a computer at home; and (5) doing other leisure activities. Within these 5 domains, values over 12 h/day were truncated to 12 h to avoid unrealistic values. Total time spent sitting on a workday and on a non-workday were calculated by summing the time reported in every domain. These totals were truncated to 16 h/day. Time spent sitting at work on a workday was used to get the daily sitting time at work. The WSQ has acceptable reliability (ICC = 0.63) and validity against objectively measured sitting time (*r* = 0.45) [46]. Participants were also asked to report the average hourly number of breaks from sitting while being at work [6]. This variable was dichotomized into ‘having less than 2 standing breaks per hour’ and ‘having at least 2 standing breaks per hour’.

Attitudes towards short standing breaks at the desk (6 items: healthy, feasible, disturbing to others, awkward, relaxing, time-losing) were measured using a 5-point response scale (‘completely disagree’ to ‘strongly agree’). Self-efficacy was measured by asking how certain employees were about having short standing breaks at their desk (4 items: when feeling tired/bad/tense/depressive, when colleagues don’t do this, when not being supported by their supervisors, and when being busy or having high time pressures; 5-point scale: ‘completely disagree’ to ‘strongly agree’). Social support was assessed by asking whether their colleagues and supervisors would support them when trying to have short standing breaks at their desk (2 items, 5-point scale: ‘completely disagree’ to ‘strongly agree’). All items were (re)coded into the same direction so that the highest scores were the most positive answers on each item. Cronbach’s α coefficients of internal consistency were calculated prior to computing related psychosocial items into one scale (i.e. attitudes: α = 0.74, self-efficacy: α = 0.73, social support: α = 0.94). Finally, employees’ intention to have more standing breaks at their desk was asked (‘no’ , ‘yes, I may do this in the future’ , ‘yes, I will try this in the next weeks’ , ‘yes, I will start doing this right away’) and dichotomized into ‘no intention’ (‘no’) and ‘intending to change’. The questions about the psychosocial correlates were based on previously validated questions to measure psychosocial correlates of physical activity [53]. The wording of the original questions was changed to reflect psychosocial correlates of sitting [54, 55].

#### Online acceptability questionnaire (completed after requesting the advice; used for the acceptability testing)

Based on existing questionnaires assessing the acceptability of previous computer-tailored interventions [36, 38], a self-administered evaluation questionnaire was developed. Six items (understandability, logic, length, clarity, lay-out of the questions, and ease of answering the questions) were used to evaluate the general assessment questionnaire prior to receiving the advice. All items were scored on a 5-point ordered response scale (‘1: completely disagree’ to ‘5: strongly agree’). Five questions (yes/no) assessed what participants did with the general advice (read it, printed it, saved it, discussed it with others, reread it later). Further, participants were asked how credible, interesting, relevant, practicable, long, motivating, and personal they found the general advice (7 items). All items were scored on a 5-point ordered response scale (‘completely disagree’ to ‘strongly agree’).

Participants were asked about the usefulness of receiving additional sections next to the general advice (5-point scale: ‘completely disagree’ to ‘strongly agree’). If participants did not request any other section of the advice, they were asked why (‘I intended to, but forgot about it’ , ‘no more interest’ , ‘it would take too long’ , ‘I didn’t want to’ , ‘website was too slow’ , ‘there were too many questions’ , or ‘it was too complicated’). For every section that was requested, the 7 items (5-point scale: ‘completely disagree’ to ‘strongly agree’) similar to those used for evaluating the general advice (section 1, see above) were used to evaluate the respective sections.

Finally, all participants were asked whether they intended (‘no’ , ‘yes, I may do this in the future’ , ‘yes, I will try this in the next weeks’ , ‘yes, I will start doing this right away’ , ‘I am already doing this’) to interrupt (have standing breaks) and reduce (replace sitting by periods of standing) their sitting (more), and how certain they are about succeeding in this (2 separate items, 5-point scale, ‘very uncertain’ to ‘very certain’).

### Data analyses

All analyses were performed using SPSS 21.0. Statistical significance was set at P < .05 and P < .10 was considered to be marginally significant.

#### Feasibility testing

Scores (means and standard deviations for quantitative variables, n and % for qualitative variables) of the socio-demographic variables, levels of sitting time and psychosocial correlates of sitting at work (assessed in 2012) were calculated for employees requesting the advice and those not requesting it. A binary logistic regression analysis was conducted to examine whether the socio-demographics, levels of sitting time and psychosocial correlates (predictors) could predict requesting the advice (dichotome outcome). Prior, all predictors were dichotomized (based on the median score for quantitative variables).

#### Acceptability testing

The mean scores (SD), ranging from 1 to 5, of the items were calculated or the proportions of participants who (strongly) agreed with the statement (answers ‘4’ and ‘5’) were provided to report on the acceptability of the advice.

## Results

### Study participants

A total of 179 participants received an invitation to request the advice and formed the sample for the feasibility testing (see Fig. 1). The sample for the acceptability testing consisted of 47 employees. Characteristics of the participants in both study samples are presented in Table 2. The majority of both samples were female, highly educated and full-time employed (see Table 2). Both samples reported on average over 11 h of sitting on a workday, including nearly 7 h of sitting at work. The samples reported about 8 h of sitting on a non-workday and more than half of the sample reported over 2 breaks per hour at work (see Table 2).

**Table 2:** Characteristics of employees included in the feasibility and acceptability testing

	Feasibility testing (*n* = 179)	Acceptability testing (*n* = 47)
*Socio-demographics*		
Gender: n (%) men	45 (25.1)	11 (23.4)
Age: years ± SD	41.0 ± 9.5	42.8 ± 9.4
Education: n (%) high (college or university)	127 (71.3)	37 (78.7)
Employment status: n (%) full-time employed	117 (65.4)	26 (55.3)
*Sedentary behaviour*		
Workday sitting time: hours/day ± SD	11.27 ± 2.73	11.32 ± 2.45
Non-workday sitting time: hours/day ± SD	7.65 ± 3.58	8.18 ± 3.67
Sitting time at work: hours/day ± SD	6.86 ± 1.29	6.75 ± 1.34
Breaks at work: n (%) ≥2 breaks/hour	111 (65.7)	26 (59.1)

### Feasibility testing

Table 3 presents the characteristics of employees requesting the advice (*n* = 90) and those not requesting it (*n* = 89). Education and employment status were found to be significant predictors of requesting the advice (see Table 3). Those with a high education were 2.4 times more likely to request the advice than those with low education, and those being part-time employed were 2.9 times more likely to request the advice compared to full-time employees. The influence of the level of breaks at work and attitudes towards interrupting sitting at work was borderline significant (P < .10; see Table 3). Employees with less than 2 standing breaks per hour were 2.1 times more likely to request the advice than those who interrupted their occupational sitting more than twice per hour. Employees with more positive attitudes were 2.0 times more likely to request the advice than those with less positive attitudes. Gender, age, workday sitting time, non-workday sitting time, sitting time at work, self-efficacy towards interrupting sitting, social support to interrupt sitting and intention to interrupt sitting did not predict whether the advice was requested or not (see Table 3).

**Table 3:** Characteristics of employees (not) requesting the advice and predictors of advice request (logistic regression)

	Employees not requesting the advice (*n* = 89)	Employees requesting the advice (*n* = 90)	Logistic regression analysis		
			*Odds ratio*	*95 % CI*	*P*
*Socio-demographics*					
Gender:			1.156	0.454–2.945	0.761
n (%) women^a^	66 (74.2)	68 (75.6)			
Age:			1.522	0.686–3.378	0.302
years ± SD^b^	40.1 ± 9.8	41.8 ± 9.1			
Education:			**2.447**	**1.027–5.828**	**0.043**
n (%) low education^a^	32 (36.4)	19 (21.1)			
Employment status:			**0.339**	**0.141–0.815**	**0.016**
n (%) part–time employed^a^	24 (27.0)	38 (42.2)			
*Sedentary behaviour*					
Workday sitting time:			1.098	0.470–2.566	0.829
hours/day ± SD^b^	11.1 ± 2.9	11.4 ± 2.5			
Non-workday sitting time:			1.892	0.877–4.081	0.104
hours/day ± SD^b^	7.4 ± 3.6	7.9 ± 3.6			
Sitting time at work:			0.886	0.374–2.101	0.784
hours/day ± SD^b^	6.83 ± 1.26	6.89 ± 1.33			
Breaks at work:			0.478	0.209–1.091	**0.080**
n (%) <2 breaks/hour^a^	22 (26.8)	36 (41.4)			
*Psychosocial correlates towards short standing breaks at the desk*					
Attitudes: mean ± SD^b^	4.1 ± 0.7	4.2 ± 0.6	1.966	0.837–4.618	**0.052**
Self-efficacy: mean ± SD^b^	3.9 ± 0.8	3.8 ± 0.7	0.405	0.163–1.007	0.892
Social support: mean ± SD^b^	3.2 ± 1.1	3.3 ± 1.1	1.054	0.496–2.239	0.161
Intention to change: n (%)^c^	73 (86.9)	73 (82.0)	0.476	0.168–1.346	0.338

^a^reference category is this group, ^b^reference category is group with lowest value through median value of this variable, ^c^reference category is group with no intention to change

*CI* confidence interval

Bold surfaces indicate statistically (borderline) significant associations (*P* < .10)

Based on administrator-data obtained through the website, it took employees 8.0 ± 3.6 min to complete the questions needed to obtain the first general section of the personalized advice. About one third of the sample (*n* = 35/90, 38.9 %) requested another section of the website other than the general section. Eighteen (*n* = 18/35, 51.4 %) of them completed the other sections on the same day as the general section. Sixteen participants requested 1 extra section, 4 participants 2 extra sections, 5 participants 4 extra sections, and 10 participants requested every section. Twenty-eight (31.3 %) participants ran through section 2, which took 2.6 ± 1.0 min, 20 (22.2 %) completed section 3 which took 3.3 ± 1.4 min, 12 (13.3 %) completed section 4 which took 1.1 ± 0.3 min, 16 (17.8 %) completed section 5 which took 1.6 ± 0.5 min and 19 (21.1 %) completed the action plan which took 4.1 ± 1.3 min.

While requesting the advice on the website participants reported a total of 11.7 ± 3.3 h of sitting on a workday and 7.8 ± 3.5 h on a non-workday. About 93 % (*n* = 81/87) received the message they sat too much (>8 h/day) on a workday.

### Acceptability testing

The results of the evaluation of the initial questioning are presented in Table 4. The majority found the questions logical, understandable and clear. About two thirds appreciated the length of the questionnaires and how the questions were presented (e.g. lay-out), and less than half of the sample was pleased with the ease of answering the questions (see Table 4). The majority (83 %) of the participants read the advice. Only limited numbers of employees printed, saved, discussed and reread the general advice (see Table 4).

**Table 4:** Evaluation of the initial questioning and usage of the general advice (section 1)

	mean scores (range 1–5) ± SD	n (%) employees (strongly) agreeing
*Evaluation of the initial questioning*		
Understandability	4.23 ± 0.60	43/47 (91.5)
Logical	4.23 ± 0.48	46/47 (97.9)
Too many questions	2.91 ± 0.90	17/47 (36.2)
Clarity	4.09 ± 0.59	41/47 (87.2)
Lay-out	3.62 ± 0.71	31/47 (66.0)
Ease of answering the questions	3.21 ± 1.02	21/47 (44.7)
*Usefulness of receiving several sections*		
I think it is useful to receive several sections	2.30 ± 0.91	4/47 (8.5)
*Usage of the general advice (section 1)*		
	n (%) employees reporting ‘yes’	
I read the general advice	39/47 (83.0)	
I printed the general advice	7/47 (14.9)	
I saved the general advice	21/47 (44.7)	
I discussed the general advice with others	9/47 (19.4)	
I reread the general advice later	13/47 (27.7)	

Table 5 presents how employees evaluated the general advice and the other sections. More than three-quarters found the advice in total credible, interesting, relevant and not too long. About two-thirds found it motivating and about half of the sample perceived the advice as personally written for them. About one third found the suggested strategies practicable, except for section 4 (commuting) of which the strategies were found to be more practicable (see Table 5).

**Table 5:** Evaluation of the advice: n (%) of participants who (strongly) agreed with the item

	General section (*n* = 47)	Section 2 (*n* = 28)	Section 3 (*n* = 20)	Section 4 (*n* = 12)	Section 5 (*n* = 16)	Action plan (*n* = 19)
Credible	37 (78.7)	22 (78.6)	18 (90.0)	11 (91.7)	15 (93.8)	14 (73.7)
Interesting	36 (76.6)	26 (92.9)	18 (90.0)	11 (91.7)	14 (87.5)	16 (84.2)
Relevant	33 (70.2)	24 (85.7)	15 (75.0)	10 (83.3)	14 (87.5)	17 (89.5)
Practicable	15 (31.9)	10 (35.7)	6 (30.0)	7 (58.3)	6 (37.5)	6 (31.6)
Too long	6 (12.8)	6 (21.4)	2 (10.0)	2 (16.6)	1 (6.3)	*not asked*
Motivating	29 (61.7)	17 (60.7)	13 (65.0)	8 (66.7)	9 (56.3)	13 (68.4)
Personal	24 (51.1)	14 (50.0)	10 (50.0)	7 (58.3)	10 (62.5)	9 (47.4)

About 58 % (*n* = 25/43) of the employees reported they were already frequently interrupting their sitting at work at the time the acceptability questionnaire was completed, 32.6 % (*n* = 17/43) intended to do so, and 2.3 % (*n* = 1/43) had no intention to change this. The majority (79.8 %, *n* = 34/43) of the employees were (very) certain they could interrupt their sitting at work more often. Replacing sitting by periods of standing was already done by 14.0 % (*n* = 6/43), 51.2 % was intending to do so (*n* = 22/43), and 34.9 % (*n* = 15/43) had no intention to do this. About 16 % (*n* = 7/43) reported to be ‘certain’ or ‘very certain’ they could succeed in replacing sitting by periods of standing.

## Discussion

In the present study, the development of a new theory-driven web-based, computer-tailored intervention aimed at influencing sitting at work was described. Furthermore, the feasibility of reaching employees and the acceptability of the intervention among its users was examined.

Concerning the feasibility of reaching employees with this intervention, positive findings are the relatively high response to the email invitation to receive personal advice (63 %) and the high proportion of interested individuals actually requesting the advice (80 %). In previous studies on computer-tailored physical activity advice, the initial response rates among the general population were 79 % when recruiting via telephone [59], 46 % when recruiting face-to-face [60], 6 % when recruiting via flyers [60] and 6 % when recruiting via general practitioners [38]. Actual advice request was also lower for these previous web-based computer-tailored interventions on physical activity (46–53 %) [59, 61] compared to the present study. Further research should confirm whether recruiting in workplaces via email, without any telephone or face-to–face contact, is indeed most effective in reaching employees for web-based, computer-tailored interventions. The present study group could possibly be a sample of already engaged employees as they previously participated in another study. Because of the select sample here, the actual number of employees open to participate and receive advice may be overestimated.

It was also encouraging that men and women, young and old individuals, those with low and high sitting levels, as well as those who scored low and high for the psychosocial correlates (self-efficacy, social support and ‘intention to change’) towards interrupting sitting at work visited the website. In a computer-tailored physical activity program, more women than men and more adults of medium socio-economic status (SES) than adults of low SES participated in the program [62]. Here, advice request was however predicted by two socio-demographic variables (education and employment status), one behavioural variable (interruption in sitting) and one psychosocial correlate (attitudes). The fact that employees with a college or university degree were more likely to request the advice is not surprising as highly educated individuals may have more knowledge about and interest in health-related behaviours [63]. It is also possible that higher educated individuals are more digital media savvy or have access to the internet through more devices (e.g. laptop, tablet, smartphone), however most employees have computer access at work. Therefore everyone was able to request the advice. Still, particular attention is needed to reach more low educated individuals in the future. Especially as some may argue that access, affordability, usability and appropriateness of e-health interventions and digital health literacy may be lower among vulnerable or underserved groups, which may lead to increased health inequalities [64]. Maybe when more attention in the media [58] is directed towards the damaging health consequences of too much sitting, this group will also be interested in receiving advice. For physical activity for example, media attention has led to increases in population awareness and knowledge about this public health problem [65].

In the present study, full-time employees were less likely to request the advice compared to part-time workers, so it may be that ‘time’ was an issue. Focus group interviews among employees and managers also revealed that productivity concerns and loss of time were barriers for the implementation of intervention strategies to reduce or interrupt sitting at work [58]. Nevertheless, as full-time employees should be reached in the future, special attention to this group is also needed. Maybe it should be explicitly stressed that requesting and applying the advice is not time-consuming. The time investment to request the general advice (section 1) was no more than 8 min. For the other sections, no more than 4 min was needed to complete the assessment questionnaires. However, it is not clear whether only this amount of time was needed to complete the questionnaires properly or whether participants rushed through the questions to get the advice as quickly as possible.

Further, there was a trend that those who had less than 2 standing interruptions per hours were more likely to request the advice, which is positive. Finally, those with positive attitudes towards interrupting sitting were more likely to request the advice. A cross-sectional study among Australian employees showed that those who perceived more advantages of sitting less at work, reported more sitting at work [55]. This would mean that the group for whom the intervention is intended (employees with high and prolonged occupational sitting time) was indeed reached, which is promising. However, Australian part-time employees reported less total and work-related sitting time than full-time employees [66], making the latter group an at-risk group. So, in the future, also those with less positive attitudes and full-time employed individuals should be reached, which may also be achieved with more (media) attention for the concept of sitting too much [58].

Regarding the acceptability of the advice, the assessment questionnaire prior to receiving the advice was evaluated positively in terms of understandability, logic, length, credibility and lay-out, however attention is needed concerning the ease of answering the assessment questionnaires. The questions were based on validated instruments, however sitting may be an automated and habitual behaviour [67], which may be hard to recall.

Further, while the authors initially assumed it would be a strength to split up the advice into separate sections to be requested when suitable for the users, only 8.5 % of the employees thought it was useful to provide the advice in separate sections. No additional questions were asked about the reason for this response. It was assumed that the freedom of choice in whether and when to request additional sections would support an individual’s feeling of autonomy, one of the three *SDT* basic needs allowing an individual to act out of autonomous motivation [42, 43]. In addition, providing all information at once would make the assessment questionnaire prior to receiving the advice very long (a total of maximum 116 questions would have to be completed) and the advice itself would also be much longer (while the length of the advice in its current format was evaluated positively). As such, it’s likely that even fewer users would read the (whole) advice. Still, it should be acknowledged that only 39 % requested other sections of the advice. More efforts are needed to motivate people to request the other sections, maybe by stressing the difference from the general section and the additional value of the sections. Perhaps the number and frequencies of the reminder emails should be increased as well. Alternatively, the different parts of the advice could be released on a weekly basis, rather than all at once. However, this undermines the principles of the *SDT* [42, 43], as the users have no autonomy in deciding when to request other sections. In addition, a structured weekly release runs the risk of few people returning to the website on multiple occasions. In the current study, about half of the participants (49 %) visited the website on more than one occasion.

It is also not promising that only 21 % of the users completed an action plan, as the *SRT* assumes that transforming implementation-intentions into specific actions (through an action plan) results in behaviour change [56, 57]. Nonetheless, the action plan scored the highest on relevance and motivation among those completing the acceptability questionnaire. In the future, it should be good to stress the usefulness of an action plan to employees willing to change their behaviour. In other studies, the proportion of users completing an action plan was not reported, but the acceptability of it was also found to be high [36, 37]. Future studies can test whether the effects in behaviour change are indeed larger in employees completing an action plan compared to those who did not.

Concerning the use of the general advice (section 1), 83 % of the employees reported they read it and 20 % discussed the advice with others. This is lower compared to other computer-tailored physical activity (96–100 % read it, 59–64 % discussed it with others) and step (96 % read it, 42 % discussed it with other) advice [37, 38]. Today, physical activity promotion programs, such as the 10,000 Steps, are well-known and popular in the general population, which does not yet apply for the ‘sitting-too-much-is-bad-for-you’-concept in Flanders [58]. It is not encouraging that only one out of five interacted with others about the advice, as group coherence and social support have been expressed as essential in order to transform challenging strategies into action and goal realization [68]. One would expect that the possibilities for peer support at work would increase through this type of intervention, however, this was not the case, maybe because of the fact that employees individually requested the advice. Discussing the advice with others could also overcome barriers, such as the awkwardness of standing or the fear of disturbing others while standing, which were reported by employees and executives in focus group interviews [58].

Overall, the majority of the sample evaluated the advice sections as credible, interesting and relevant, which is comparable to previously developed computer-tailored physical activity and step advice [37, 38]. The low scores on practicability (except for section 4, sitting during commuting) may be due to the fact that interventions to influence sitting are fairly new. Employees may perceive the suggested strategies as hard to apply because they are not familiar with this concept, nor are their managers. The lowest applicability score was found for section 3 (replacing sitting at work by standing). This was also reflected in the ‘intention to change’ scores reported after receiving the advice, as only 14 % was already replacing sitting by standing and only 16 % was certain they could succeed in starting/continuing this. In contrast, more than half reported (58 %) they were already frequently interrupting their sitting at work and another 33 % intended to do so. About 80 % also believed they were able to continue/accomplish this. These important findings suggest that interrupting sitting is more achievable to implement than replacing longer periods of sitting by standing at work. The health promotion message “*good STUFF*” (Stand Up For Fitness) suggested by Rutten et al. (2013) [52] is also mainly encouraging interruptions in long sitting periods by short breaks. However, the majority of interventions in this area have focussed on reductions in total sitting time [16–23]. For example, the intervention “*Stand Up, Sit Less, Move More*”, encourages to use workstations in order to have equal amounts of sitting and standing time, to stand up at least every 30 min, and to increase incidental physical activity [19, 23]. The latter intervention showed promising short-term effects for sitting and standing outcomes. Further research should investigate which strategies to influence sitting at work are most feasible, acceptable and effective among employees.

The overall strength of this research is the web-based approach of influencing sitting at work. This computer-tailored approach was theory-driven and the advice was based on current evidence available in the literature. This type of intervention has not been used or tested for feasibility and acceptability before. Second, the assessment questionnaires were based on or similar to existing validated questionnaires [40, 46–48, 53–55] and the questionnaire evaluating the acceptability of the advice was also based on existing measures [36, 38]. There are, however, some limitations to take into account. First, the mainly female and highly educated sample was recruited within only one company that had already participated in another study and may consequently be more interested in health-related behaviours and research. Furthermore, the ‘intention to change’ towards short standing breaks was also high. For these reasons, this sample may not be representative for the broader working population, which compromises generalizability of the findings. In the future, feasibility and acceptability studies should be done in several companies, covering a large variety of employees. Second, all data were self-reported which may cause recall and social desirability biases. The use of objective measures, such as accelerometers or inclinometers, could limit these biases. The self-reported sitting at work of the present sample was on average circa 6.8 h/day, which is in line with a previous study using self-reports in an Australian convenience sample (6.5 h/day) [46]. However, this is higher than occupational sitting reported in a Danish population-based sample (4.4 h/day) [69] and in a sample of Australian workers (3.3 h/day) [70], and higher than objectively measured occupational sitting time in Australian office employees (4.3 h/day; 5.6 h/day) [19, 71]. Third, qualitative methods were not applied during intervention development, while including input from the target group, could have enhanced the intervention. For example, the perceived uselessness of separating the advice in several sections could have been discovered earlier. Also for the feasibility and acceptability testing, a mixed methods approach, including focus groups or interviews, could have resulted in more in-depth information about certain aspects, such as the high initial response rate and the relatively low proportion completing an action plan. In addition, through qualitative methods, the potential benefits of this advice in a multi-component intervention could have been discussed. Finally, for the feasibility study, the data were collected in November 2012. Some outcomes may have changed at the time the present feasibility study was conducted (February 2014). Future studies should use more recent data when predicting advice request.

## Conclusions

It is feasible to reach employees across most different socio-demographic groups with this newly developed theory-driven web-based, computer-tailored intervention aimed at influencing sitting at work. Still, more efforts are needed to reach lower educated and full-time workers. The majority of the users found the tailored advice acceptable in terms of the assessment questioning, interestingness, length, credibility and relevance of the advice. Acceptability scores were lower for the practicability of applying the advice, however most of the employees who requested the advice, reported they changed their behaviour or intended to do so. Interrupting sitting at work appeared more achievable than replacing sitting time by periods of standing. Future research should investigate the effects of this intervention on correlates of sitting (e.g. knowledge, attitudes) and on reductions and interruptions of sitting in a pre-post-test randomized controlled trial.

## Abbreviations

CI: Confidence interval
SB: Sedentary behaviour
SD: Standard deviation
SDT: Self-Determination Theory
SRT: Self-Regulation Theory
TPB: Theory of Planned Behaviour
WSQ: Workforce Sitting Questionnaire
